# The first family with adult osteogenesis imperfecta caused by a novel homozygous mutation in *CREB3L1*

**DOI:** 10.1002/mgg3.823

**Published:** 2019-06-17

**Authors:** Ferdy K. Cayami, Alessandra Maugeri, Sanne Treurniet, Eva D. Setijowati, Bernd P. Teunissen, Elisabeth M.W. Eekhoff, Gerard Pals, Sultana M. Faradz, Dimitra Micha

**Affiliations:** ^1^ Department of Clinical Genetics, Amsterdam Movement Sciences Amsterdam UMC, Vrije Universiteit Amsterdam Amsterdam The Netherlands; ^2^ Center of Biomolecular Research, Faculty of Medicine Diponegoro University Semarang Indonesia; ^3^ Department of Internal Medicine Section Endocrinology, Amsterdam Movement Sciences Amsterdam UMC, Vrije Universiteit Amsterdam Amsterdam The Netherlands; ^4^ Biomedical Department, Faculty of Medicine Wijaya Kusuma University Surabaya Indonesia; ^5^ Department of Radiology and Nuclear Medicine Amsterdam UMC, Vrije Universiteit Amsterdam Amsterdam The Netherlands

**Keywords:** CREB3L1, hereditary, osteogenesis imperfecta, osteoporosis, skeletal dysplasia

## Abstract

**Background:**

Osteogenesis imperfecta (OI) is a clinically heterogeneous disease characterized by extreme skeletal fragility. It is caused by mutations in genes frequently affecting collagen biosynthesis. Mutations in *CREB3L1* encoding the ER stress transducer OASIS are very rare and are only reported in pediatric patients. We report a large family with a novel *CREB3L1* mutation, with severe adult clinical presentation.

**Methods:**

Clinical examination was performed on the family members. Next generation sequencing was performed for the causative genes for OI. The mutation was confirmed in other family members with Sanger sequencing.

**Results:**

A novel homozygous mutation in *CREB3L1* was identified in the three affected patients. The parents and siblings who carry the mutation in heterozygous state were clinically unaffected. The three affected siblings, who were reported to have been born healthy, presented very severe progressive skeletal malformations and joint contractures but absence of common OI characteristics including blue sclerae, deafness, and dentinogenesis imperfecta. Resorption of a part of the humerus presumably associated with fracture nonunion and pseudarthrosis.

**Conclusion:**

We report a novel homozygous *CREB3L1* mutation in a large Indonesian family; the homozygous affected members have survived to adulthood and they present a more severe phenotype than previously reported, expanding the clinical spectrum of OI for this gene.

## 1 INTRODUCTION

OI patients experience numerous bone fractures as a result of bone tissue fragility. The vast majority of cases show an autosomal dominant pattern of inheritance and are mostly caused by mutations in collagen type I. The rest of the cases are autosomal or X‐linked recessive and are caused by mutations in approximately 16 genes many of which are implicated in collagen biosynthesis. One of the genes for recessive OI is *CREB3L1* (OMIM 616215) which is responsible for the production of the old astrocyte specifically induced substance (OASIS) which is a transcription factor involved in cell stress response. In the bone tissue it regulates the expression of collagen type I and matrix proteins and it influences osteoblast maturation(Murakami et al., 2009).

Different mutations in *CREB3L1* have been previously reported only in 3 OI families. In the first two families the homozygous patients presented neonatal lethality (Keller et al., 2018; Symoens et al., 2013) whereas in the third family a 11‐year‐old boy with severe bone deformities and other syndromic OI features was described(Lindahl et al., 2018). This study aims to report the fourth OI family caused by a new mutation in *CREB3L1*. The homozygous patients are the first to reach adulthood and they present severe progressive clinical features fitting classification as OI type III. This report expands on the genetic and clinical spectrum of the OI causative gene *CREB3L1*.

## 2 METHODS

Genomic DNA was isolated from the peripheral blood of the three affected patients, the parents and an unaffected sibling according to standard protocols. The quality and quantity was measured by NanoDrop spectrophotometer. DNA from patient II:5 was subjected to targeted next generation sequencing (NGS) analysis with the routine gene panel that includes the common disease‐causing genes for OI. The analysis was performed in the Genome Diagnostics Laboratory of the VU University medical centre with the Illumina sequencing platform by using a NimbleGen SeqCap EZ Human Exome Library v2.0 custom‐made enrichment kit on an Illumina HiSeq2000 sequencer. The coverage of this analysis was at least 30 times for the targeted regions. The gene panel included *COL1A1*, *COL1A2*, *ALPL*, *BMP1*, *CREB3L1*, *CRTAP*, *FKBP10*, *IFITM5*, *LEPRE1*, *LRP5*, *PLOD2*, *PLS3*, *PPIB*, *SERPINF1*, *SERPINH1*, *SP7*, *TAPT1*, *TMEM38B*, *WNT1*. For the bioinformatics analysis an established in house pipeline was used to process the NGS data including the Burrows‐Wheeler Aligner for alignment of reads, the Genome Analysis Tool Kit Lite for bam file processing and Cartagenia for selection of the disease genes. Genetic variants in these genes were then interrogated by in silico pathogenicity analyses. A putative genetic variant in *CREB3L1* was tested by Sanger sequencing in the other family members.

The patients were physically examined by a team of experienced medical professionals. Informed consent and ethical clearance were obtained from the Faculty of Medicine Diponegoro University/Dr Kariadi General Hospital and the Faculty of Medicine Udayana University/Sanglah Hospital ethical committee and Amsterdam UMC.

## 3 RESULTS

### 3.1 Clinical report

**Figure 1:**
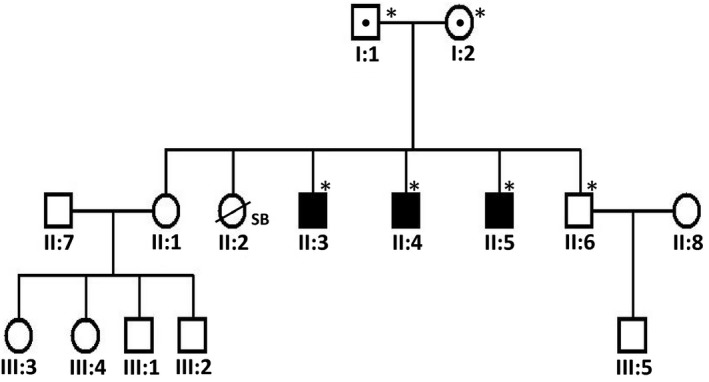
The pedigree of the described family. Affected individuals are indicated by filled symbols whereas dots indicate heterozygous carriers. Asterisks show the individuals who were tested for the c.1365del mutation in the *CREB3L1* gene

The three affected patients are part of a large family which is reported to be nonconsanguineous from the Ubud city on Bali in Indonesia (Figure 1). According to the parents the three brothers were born normal after which the disease became progressive with severe bone deformities reminiscent of OI type III, although there is no clinical record to support this. The patients have no birth certificate but are estimated to be in their thirties. They have no sign of dentinogenesis imperfecta or blue sclerae and they are intellectually competent. The parents and siblings of the patients were examined and found to be normal with regard to scleral color, dental abnormalities, joint hyper‐extensibility, auditory ability, as well as bone fractures and dysplasia.

**Figure 2:**
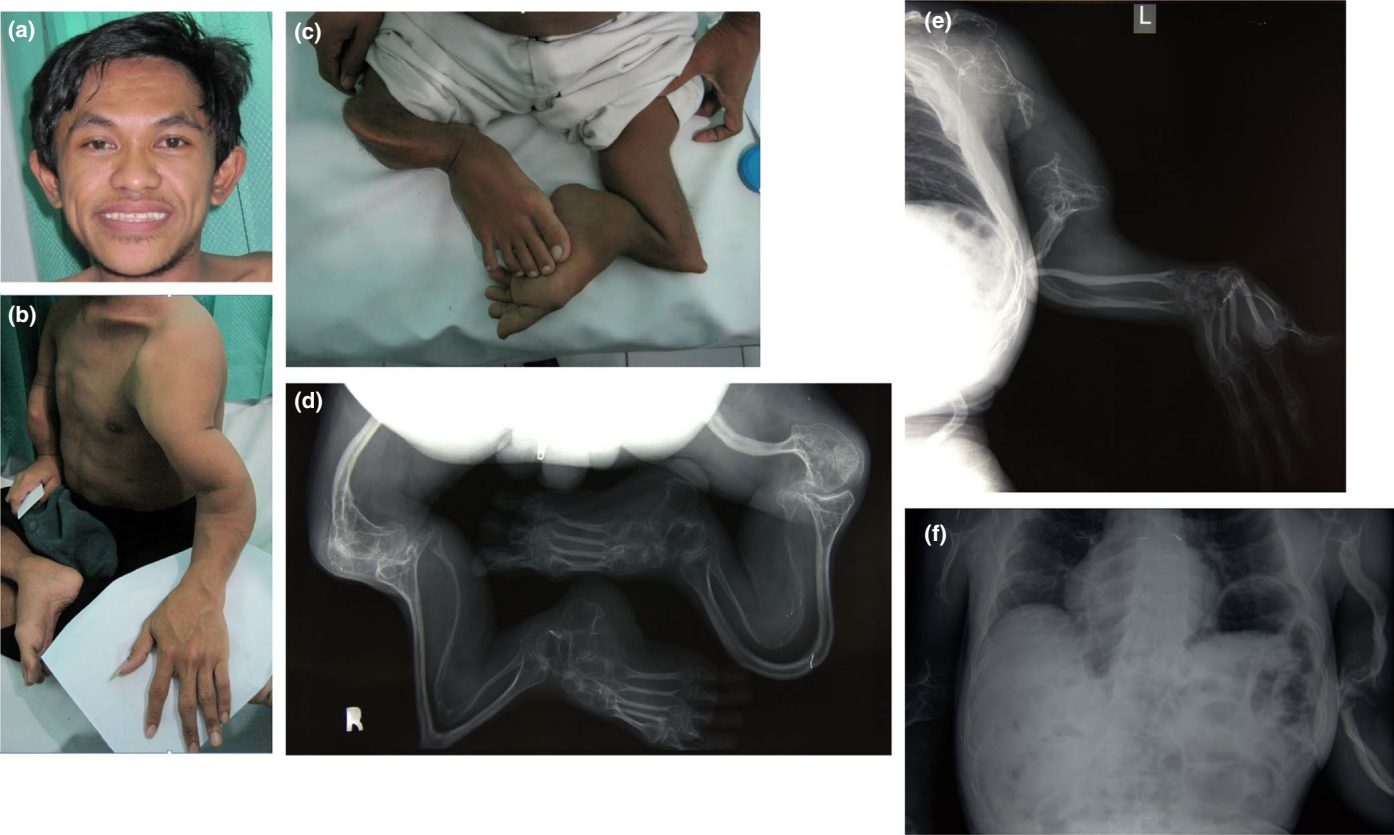
Clinical characteristics of the three affected brothers. (a) Facial features in patient II:5 showing normal sclerae and teeth. (b) Dysmorphic features in the left arm of patient II:5; the size of both hands is normal. (c) Severe bending of the femur, tibia, and fibula in patient II:5. (d) Radiological image in patient II:5 showing the skeletal malformations in the lower extremity. (e) Radiograph of left arm of patient II:5 revealing extensive resorption of the humeral shaft. (f) Radiograph of thoracic cavity in patient II:4 showing the appearance of wavy ribs.

All three OI affected siblings started having bone fractures before 4 years old (Figure 2). Bone fractures have taken place in both legs, arms, and ribs; average recovery time is 3–12 months. Joint contractures have been severe and progressive. They have very short stature and require ambulation with a wheelchair. There is exaggerated posterior bending of the tibia which happened without fracture and was painful. Patient II:4 underwent an unsuccessful rodding procedure to straighten the tibia, after 1 year the rod was removed because it was bent.

The radiological findings of the three brothers are almost identical. The skeletons in general appeared to be osteopenic. The upper extremity is characterized by distortion of the humeral head on both sides and resorption. In all three affected brothers there is resorption of a part of the humeral shaft, likely related to prior fractures resulting in nonunion. In patient II:3 there is resorption of the left humerus, in patient II:4 of the right humerus and in patient II:5 it is bilateral. There is bending of the ulna and radius on both arms. Also in the lower extremities there is severe bending of the femur, tibia, and fibula. The ribs (costae) show a wavy aspect and as far as visible there are no signs of previous fracture. The skeleton of the hands and feet is unaffected.

### 3.2 Genetic analysis

**Figure 3:**
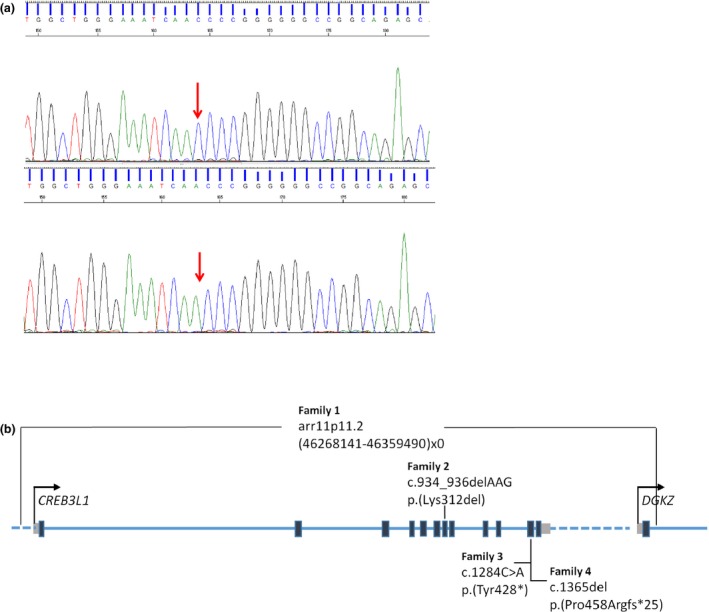
(a) Chromatogram of Sanger sequencing. The arrow shows the c.1365del mutation in the *CREB3L1* gene (NM_052854.3), indicating the deletion of a cytosine in patient II‐5. (b) Schematic presentation of the CREB3L1 protein showing the location of the reported mutations in four families with OI. The diagram presents the 12 exons of this protein and the first exon of the gene *DGKZ*. Black boxes indicate exons, grey boxes indicate 5′and 3′untranslated regions whereas the dotted line indicates intragenic regions

Analysis of the targeted NGS panel for the common OI‐related genes yielded a homozygous pathogenic variant (c.1365del) in exon 11 of the gene *CREB3L1* (RefSeq NM_052854.3) in patient II:5. The mutation was confirmed by Sanger sequencing in patient II:5 and the two other affected patients of the family (II:3 and II:4) whereas it was not found to be present in their unaffected brother II:6 (Figure 3a). This mutation leads to the deletion of a nucleotide which produces a frameshift effect starting at codon Pro458 (p.Pro458Argfs*25). As a result the reading frame for 25 codons is altered downstream of the mutation at the end of which a stop codon is formed. Considering that the newly formed stop codon is further than 55 nucleotides upstream of the last exon‐exon junction, there is strong prediction that the produced mRNA will be subjected to nonsense mediated decay (NMD) by which the mRNA is subjected to degradation leading to absence of protein expression. However, this was not possible to be confirmed experimentally given the inability to acquire patient cells. In case that the mRNA would escape nonsense mediated decay, a truncated protein will be produced with part of exon 11 and whole of exon 12 missing (Figure 3b). In this case most of the protein would remain intact. This mutation has not been reported before in any population databases including dbSNP, 1000Genomes, ExAC, GnomAD, and ClinVar. The unaffected parents were both found to be positive for the mutation in heterozygous state. Segregation is consistent with the described recessive inheritance pattern of *CREB3L1*.

## 4 DISCUSSION

In the first two reported families mutations in *CREB3L1* were regarded as a cause for perinatal lethal OI (Symoens et al., 2013)(Keller et al., 2018). All homozygous patients exhibited clinical features of severe skeletal dysplasia and multiple bone fractures; these pregnancies were medically terminated. In the first family a patient with severe OI characteristic bone deformities survived until 9 months of age; although he was not genetically tested, homozygosity for the mutation is expected (Symoens et al., 2013). In both families the parents and siblings were found to have the mutation in heterozygous state and they showed a mild phenotype with variable OI features such as blue sclerae, osteopenia, and bone fractures. Dental abnormalities were absent in both families. Given the presence of a very severe phenotype in homozygous state and of milder symptoms in heterozygous state, inheritance in these families can be described as incomplete dominance. In the recently reported third family, the pediatric patient has survived up to 11 years old (Lindahl et al., 2018). This patient experienced multiple fractures *in uterus* and later in his life. Although skeletal deformity was prominent at birth, he received from a young age treatment with bisphosphonates and rodding surgery which is expected to have improved clinical presentation. He has some tooth agenesis. Interestingly, the heterozygous parents and siblings had no OI symptoms. Homozygous patients of our family have survived to adulthood, into their thirties. They have very severe OI which is described to be progressive; all three of them were reported to have been born healthy. They had no treatment with bisphosphonates and a rodding procedure in the tibia of patient II:4 was short and unsuccessful. They have exaggerated bending of the radius, ulna, tibia and fibula. In all of them the humeral shaft is partly resorbed and weak. Similarly to the third family, the tested heterozygous parents and sibling are healthy which clearly indicates a recessive pattern of inheritance.

In order to attempt explanation of the high phenotypic variation in the four families, the effect of the mutation must be considered. CREB3L1 resides in the endoplasmic reticulum (ER) membrane and in response to stress, the N‐terminus is cleaved, which translocates to the nucleus to regulate expression of genes including *COL1A1*. In the first family a deletion of the whole gene caused absence of CREB3L1 expression. The deletion also encompassed the first exon of *DGKZ* which may prevent its expression; downregulation of DGKZ can increase osteoclast differentiation and resorption (Iwazaki et al., 2017). In the second family, the mutation c.934_936delAAG resulted in the *in frame* deletion of one amino acid in a conserved DNA binding domain which abolishes this function. In the third and our family, both mutations (c.1284C>A and c.1365del respectively) in exon 11 resulted in a premature stop codon; in the third family this was shown to lead to CREB3L1 downregulation due to NMD. Because of absence of patient material this could not be confirmed in our family; nonetheless, it is predicted. However, NMD efficiency can vary which means that a residual amount of a truncated protein may be produced. Considering that the stop codon is found in exon 11 of a total 12 exons, this would produce a largely intact and possibly functional protein. This could account for the milder phenotype in these two families. The patients in our family have more severe OI than the boy in the third family; it is unclear to which extend the milder symptoms in the boy can be attributed to his medical treatment or how similar was the phenotype of our patients at his age.

This report adds to the mutation and phenotype spectrum of the rare OI gene *CREB3L1*. Patients with this mutation can survive to adulthood despite the very severe bone malformation symptoms of their disease.

## CONFLICT OF INTEREST

The authors declare no conflict of interest.
